# Dual RNase activity of IRE1 as a target for anticancer therapies

**DOI:** 10.1007/s12079-023-00784-5

**Published:** 2023-09-18

**Authors:** Sylwia Bartoszewska, Jakub Sławski, James F. Collawn, Rafał Bartoszewski

**Affiliations:** 1 Department of Inorganic Chemistry, Medical University of Gdansk, Gdansk, Poland; 2 Department of Biophysics, Faculty of Biotechnology, University of Wrocław, F. Joliot-Curie 14a Street, 50-383 Wrocław, Poland; 3 Department of Cell, Developmental, and Integrative Biology, University of Alabama at Birmingham, Birmingham, AL 35233 USA

**Keywords:** ER-stress, RIDD, Basal RIDD, XBP1, UPR, IRE1α

## Abstract

The unfolded protein response (UPR) is a cellular mechanism that protects cells during stress conditions in which there is an accumulation of misfolded proteins in the endoplasmic reticulum (ER). UPR activates three signaling pathways that function to alleviate stress conditions and promote cellular homeostasis and cell survival. During unmitigated stress conditions, however, UPR activation signaling changes to promote cell death through apoptosis. Interestingly, cancer cells take advantage of this pathway to facilitate survival and avoid apoptosis even during prolonged cell stress conditions. Here, we discuss different signaling pathways associated with UPR and focus specifically on one of the ER signaling pathways activated during UPR, inositol-requiring enzyme 1α (IRE1). The rationale is that the IRE1 pathway is associated with cell fate decisions and recognized as a promising target for cancer therapeutics. Here we discuss IRE1 inhibitors and how they might prove to be an effective cancer therapeutic.

**Graphical abstract:**

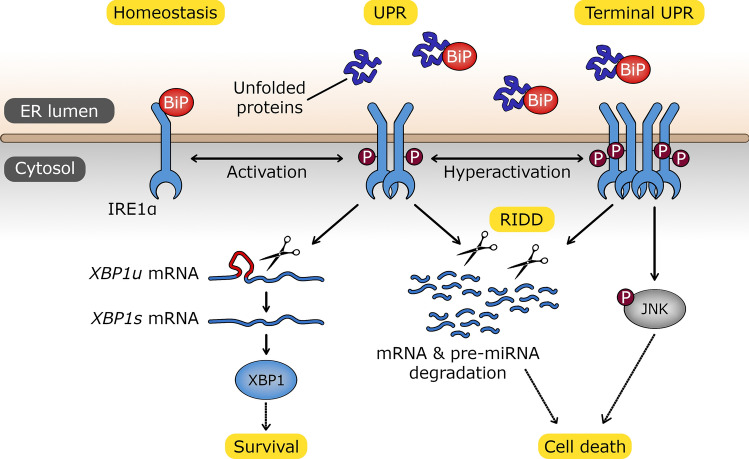

## Introduction

**Fig. 1:**
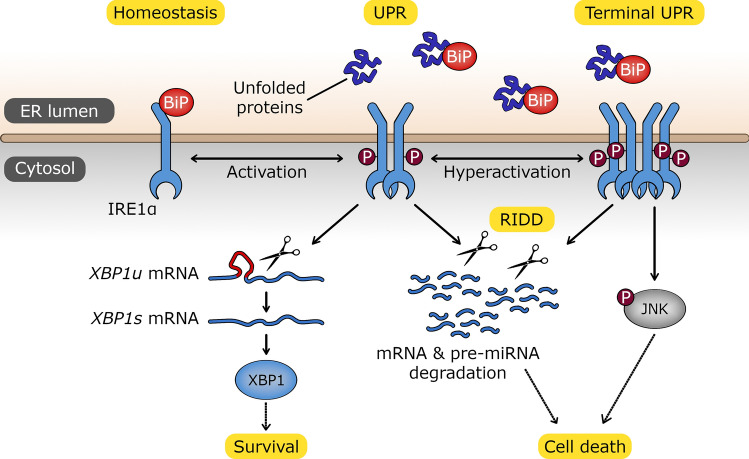
Schematic representation of the cell fate downstream consequences of ER stress-induced IRE1 activation that includes (i) the proadaptive XBP1s-dependent transcriptional signaling and (ii) the apoptotic RNA degradation (RIDD) and JNK pathway activation. The stabilization of the oligomeric form of IRE1 leads to continuous UPR activation

During tumor development and progression, transformed cells adapt to their increased demands on protein and lipid production required for rapid growth by enhancing endoplasmic reticulum (ER) function and expansion (Bartoszewska et al. 2022; Madden et al. 2019). To accomplish this, cancer cells take advantage of an adaptive multifunctional signalling pathway called the unfolded protein response (UPR) (Madden et al. 2019). The function of this pathway is to protect cells from an accumulation of unfolded or misfolded proteins in ER. UPR does this by activating three ER transmembrane sensors, inositol-requiring enzyme 1α (IRE1), protein kinase RNA-like endoplasmic reticulum kinase (PERK) and activating transcription factor 6 (ATF6). The function of UPR and these signalling pathways is to restore proper ER function and thus promote cell survival. If cellular homeostasis is difficult or potentially impossible to restore, cell death occurs usually through apoptosis (Madden et al. 2019; Karagoz et al. 2019; Almanza et al. 2019). Interestingly, cancer cells avoid this UPR transition to apoptosis, and therefore strategies that inhibit the survival pathways have become an attractive target for anticancer therapies (Bartoszewska et al. 2022; Lhomond et al. 2022; Balkwill et al. 2012). Although all three UPR sensors provide appealing therapeutic candidates, recently IRE1 activity has been a major focus since elevated levels of IRE1 are associated with poor cancer prognosis (Bartoszewska et al. 2022; Lhomond et al. 2022). IRE1 splices an inactive unspliced form of XBP1 to generate a highly active prosurvival transcription factor, spliced *XBP1* (*XBP1s*). XBP1s’s function is to enhance the expression of ER-resident chaperones and to promote ER expansion (Marchant et al. 2010; Bartoszewska et al. 2019). IRE1 also cleaves other mRNAs localized to the ER membrane through regulated IRE1-dependent decay (RIDD) (Hollien et al. 2009). IRE1 activity can serve both adaptive and apoptotic branches of UPR (Bartoszewska et al. 2022; Martinez-Turtos et al. 2022) **(**Fig. 1**)**, and therefore inhibiting IRE1 activity has consequences for both branches of UPR, which makes targeting IRE1 in anticancer therapies quite challenging (Martinez-Turtos et al. 2022).

## The UPR

ER membranes prevent direct coupling between mRNA translation and protein folding making this organelle crucial for transmembrane and secretory proteins maturation (Bravo et al. 2013). However, although maintaining ER homeostasis is an absolute requirement for proper cellular function, numerous endogenous and exogenous insults can deregulate ER function and lead to ER stress. ER stress results from an accumulation of unfolded or incompletely folded proteins in the ER lumen and that requires elevated chaperones levels (Almanza et al. 2019). Hence, to improve ER protein folding, glucose-regulated protein 78 (GRP78 also known as BiP (binding immunoglobin protein)) is released into the ER lumen from three ER transmembrane sensors: PERK, IRE1α, and ATF6 (Almanza et al. 2019; Hetz 2012). With BiP removal, these three sensors become activated and launch the UPR.

In other words, during basal conditions BiP is associated with the ER stress sensors, keeping them inactive. Under stress, however, BiP is released into ER lumen to bind misfolded peptides, and this enables activation of the ER stress sensors and consequently initiation of UPR (Hetz et al. 2020). Released BiP allows ATF6 to exit the ER and traffic to Golgi apparatus where it is cleaved by site 1 and site 2 proteases, yielding a nuclear-targeted transcription factor ATF6f (p50) (Schroder and Kaufman 2005; Ye and Koumenis 2009; Haze et al. 1999).

IRE1 and PERK share a similar mechanism of activation (Zhou et al. 2006; Carrara et al. 2015) and loss of BiP permits both kinases to self-associate and undergo trans-autophosphorylation to become active. Activated PERK is able to phosphorylate the alpha subunit of the eukaryotic initiation factor 2 (eIF2). eIF2 is a GTP-binding protein necessary for the cap-dependent mRNA translation since it delivers the initiator methionyl-tRNA to the ribosome. Although the phosphorylated eIF2 inhibits the activity of its own guanine nucleotide exchange factor, this leads to the reduction of global rates of protein synthesis (Baird and Wek 2012). This also allows a subset of mRNAs to be translated more efficiently, including growth arrest and DNA damage inducible protein (*GADD34*) that in complex with G-actin and protein phosphatase 1 (PP1) dephosphorylates eIF2 to restore normal rates of translation when the ER stress is mitigated (Novoa et al. 2001). Other mRNAs translated more efficiently are the proapoptotic CCAAT/enhancer binding homologous protein (*CHOP*) and activating transcription factor 4 (*ATF4*) (Rutkowski and Kaufman 2003). PERK activation and its downstream effects have been termed the integrated stress response (ISR) (Calabrese et al. 2007; Rzymski and Harris 2007; Blais and Bell 2006; Herman 2006).

IRE1 is a type I transmembrane ER resident protein that contains two enzymatic activities, serine/threonine kinase and endoribonuclease (RNase) activities. Kinase activity’s only function is for autophosphorylation. Trans-autophosphorylation activates its endonuclease domain that splices the mRNA transcript of X-box binding-protein (XBP) transcription factor into a transcriptionally active isoform (*XBP1s*) (Yoshida et al. 2001) and the endonuclease activity degrades a subset of mRNAs to relieve the ER load of newly translated proteins. This latter function is termed IRE1-dependent decay (RIDD) (Han et al. 2013; Maurel et al. 2014).

**Fig. 2:**
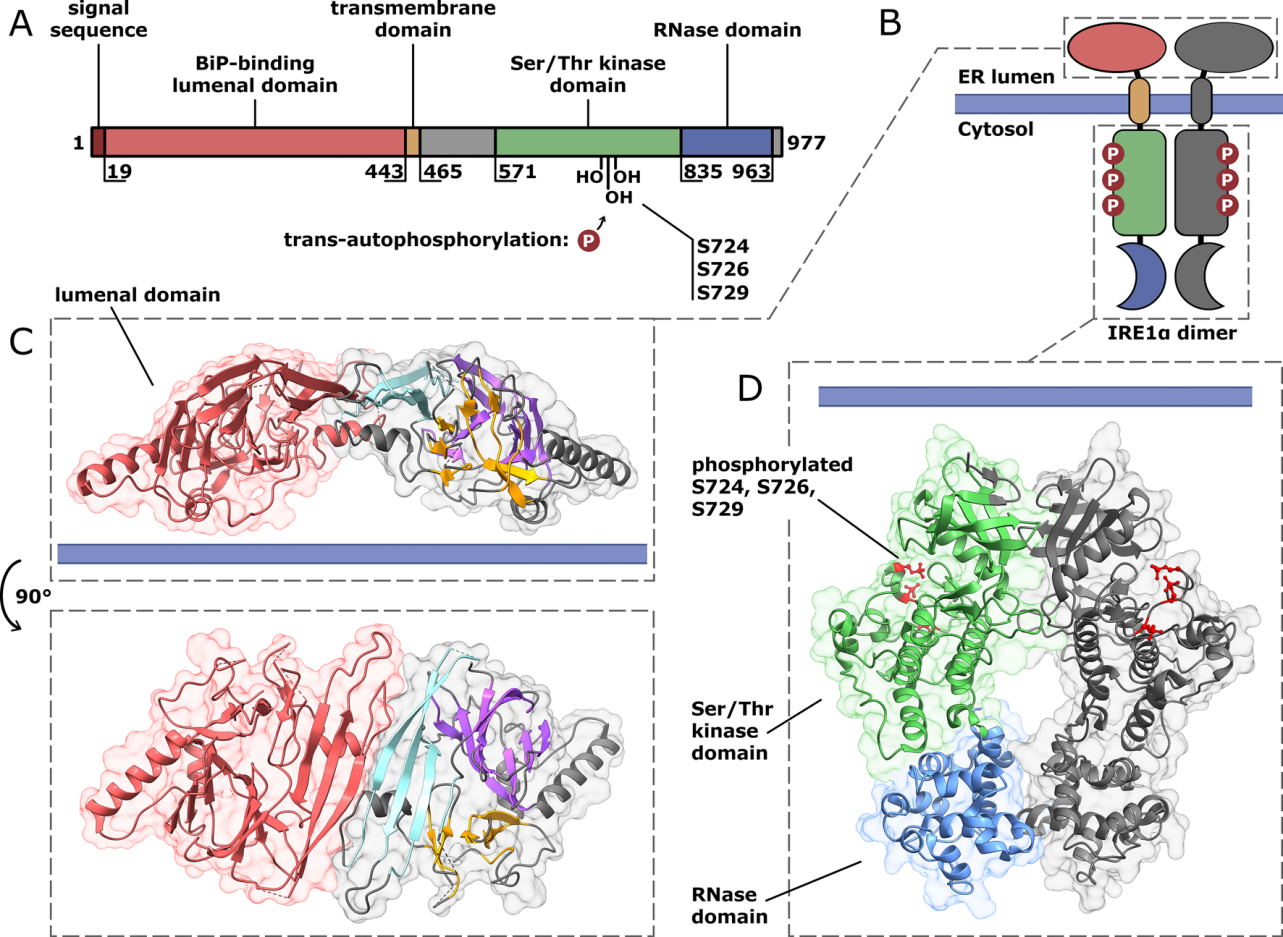
Domain structure of human IRE1α. **A** IRE1 contains an 18-amino acid N-terminal signal sequence directing the protein to the endoplasmic reticulum (ER). Lumenal domain of IRE1 is a binding site for binding immunoglobin protein (BiP). Transmembrane domain is a single-pass peptide and is followed by cytoplasmic part, consisting of two catalytic domains: serine/threonine protein kinase and endoribonuclease (RNase). The only known substrates of IRE1α kinase activity are S724, S726, and S729 residues of IRE1 itself. **B** The schematic topology of IRE1 dimer. **C** The crystal structure of the IRE1α lumenal domain dimer (PDB ID: 2HZ6), the side and top view. The IRE1 lumenal domain comprises a triangular plate of three β-sheet clusters (colored in purple, orange, and cyan) forming an extensive dimerization interface.** D** The crystal structure of the cytoplasmic part of the IRE1 dimer (PDB ID: 6W3C). Phosphorylated S724, S726, and S729 residues are depicted in red

As shown in Fig. 2A, the domain structure of IRE1α consists of a signal sequence, a BiP-binding domain, a transmembrane region, a serine/threonine kinase domain, and the RNAse domain. (Liu et al. 2003, 2002). The ER lumen exposed domain of IRE1 provides binding site for BiP (Fig. 2B), and it’s connected thorough a single-pass transmembrane segment with the catalytically active cytoplasmic region of this protein (Fig. 2B) (Zhou et al. 2006). Upon BiP dissociation, a triangular plate-like structure within this domain is formed by three β-sheets clusters, and this provides a scaffold for IRE1 dimerization (Fig. 2C) (Zhou et al. 2006). Starting from ER membrane, the cytosolic part of IRE1 contains serine/threonine protein kinase domain that is responsible for the trans-autophosphorylation is located within its serine residues (S724, S726, and S729) and this is followed by the RNAse domain (Fig. 2D) (Ferri et al. 2020).

The main function of UPR is to adjust the cellular signaling pathways in order for stressed cells to survive an insult and restore normal ER function (Bartoszewska et al. 2022; Gebert et al. 2021). The consequence of this is that the ER protein influx is reduced, and misfolded proteins are degraded by ER-associated degradation (ERAD) (Ruggiano et al. 2014; Bartoszewski et al. 2016). ATF6f and XBP1s promote ER membrane biosynthesis, increased folding capacity of the ER and increased expression of genes involved in ERAD and N-glycosylation (Baird and Wek 2012; Gebert et al. 2021; Zhang and Kaufman 2004; Mori et al. 1996; Yoshida et al. 2000). Furthermore, along with these transcription factors, ATF4 prevents cell death by increasing pro-survival and antiapoptotic signaling (Wortel et al. 2017).

If cells are exposed to persistent and intense ER stress that prevents effective restoration of ER homeostasis, the UPR triggers an intrinsic apoptotic pathway. Although the molecular basis of this cell fate switch is still poorly understood, it is mediated by all 3 UPR sensors. This includes the accumulation of CHOP (PERK and ATF6f) and the activation of the Janus N-terminal kinase (JNK) (IRE1) (Hollien et al. 2009; Obiedat et al. 2019; Pozzi et al. 2016; Carlesso et al. 2019). Furthermore, UPR cell fate decisions are also determined by a network of apoptotic factors such as growth arrest and DNA damage-inducible alpha (GADD45A), p53 upregulated modulator of apoptosis (PUMA), and phorbol-12-myristate-13-acetate-induced protein 1 (PMAIP1, also known as NOXA) (Bartoszewska et al. 2022; Gebert et al. 2021, 2018; Reimertz et al. 2003; Gupta et al. 2012; Wang et al. 2009; Rosebeck et al. 2011). Finally, in addition to the transcriptional and translational mechanisms involved in the UPR, posttranscriptional miRNA-based modulation of both survival and apoptotic activities of this pathway have been reported (Bartoszewska et al. 2013, 2017, 2019, 2022; Byrd and Brewer 2013, 2011; Kim and Croce 2021; Mukherji et al. 2011; Maurel and Chevet 2013; Cheung et al. 2008).

## Cancer cells benefit from the UPR

Given the critical role of UPR in both maintaining ER function and determining cell fate decisions, it is not surprising that this complex pathway accompanies many human diseases including metabolic disorders, neurological disorders, and cancer. In cancer cells, rapid proliferation requires increased lipid and protein synthesis (Chen and Cubillos-Ruiz 2021; Babour et al. 2010), whereas solid tumor microenvironments are often limiting in both nutrients and oxygen (Lane et al. 2020). Furthermore, the oncogenic transformations and chromosomal abnormalities can increase the fraction of misfolded proteins in ER lumen. Nevertheless, despite the fact that cancer cells are often exposed to persistent ER stress, they benefit from deregulation of the prosurvival UPR signals. For example, ATF6-related pro-survival signaling was reported in gastric tumors (Jeong et al. 2015) and mutant p53 cancer cells (Sicari et al. 2019), whereas both XBP1s and IRE1 allow for the cells to tolerate high levels of MYC proto-oncogene and to avoid cell death (Tameire et al. 2015; Sheng et al. 2019; Zhang et al. 2020; Shajahan-Haq et al. 2014).

Multiple myeloma cells frequently overproduce immunoglobulins subunits and have become the target of the first clinical UPR-targeted therapy to impair ERAD with the proteasome inhibitor, bortezomib (Bross et al. 2004; Meister et al. 2007; Kane et al. 2003; Obeng et al. 2006). Interestingly, bortezomib treatments lead to ER stress that is above the limits these cancers cells can adapt and therefore this leads to cell death and clinical improvement (Wang et al. 2009; Bross et al. 2004; Auner et al. 2013). That being said, cancer stem cells survive these treatments due to their lower translational needs and eventually mutate the proteasome proteins to acquire drug resistance that leads to the cancer relapse (Oerlemans et al. 2008; Balsas et al. 2012; Uyuklu et al. 2007). Furthermore, proteasome inhibition was also promising in vitro against glioblastoma multiform cells (Yoo et al. 2017; Lee et al. 2017). Currently, besides bortezomib, two other proteasome inhibitors are in clinical use (carfilzomib and ixazomib). In contrast, indirect approaches to limit ERAD have failed clinical trials due to off-target effects or poor specificity (Marciniak et al. 2022; Zhai et al. 2021; Ding et al. 2022; Alrasheed et al. 2021).

PERK activity has also been utilized by cancer cells to adjust the proliferation-related rate of protein synthesis to modulate growth rates (Atkins et al. 2013; Bi et al. 2005). Furthermore, in glycolytic cancer cells, PERK activity induces carbonic anhydrase 9 (CA9) and thus prevents cells acidosis (Beucken et al. 2009). Whereas ATF4 and CHOP enhance defense against redox disturbance and reactive oxygen species (ROS) (Rouschop et al. 2013; Harding et al. 2003; Baker et al. 2012; Melber and Haynes 2018; Shpilka and Haynes 2018; Loinard et al. 2012**).** In agreement with these findings, PERK and ATF4 have been shown to protect glioblastoma cells from radiotherapy and hypoxia-related oxidative damage (Mujcic et al. 2013; Mudassar et al. 2020). Both PERK and ATF4 were also shown to support autophagy in many hypoxic and nutrient-deprived tumors and cell lines (Rzymski et al. 2010; Rouschop et al. 2010; Singleton and Harris 2012**).** Interestingly, in some tumors CHOP expression was modest and did not accelerate cell death (Koumenis et al. 2002**),** whereas in the others hypoxic tumors accumulation of CHOP resulted in increased ROS production (Marciniak et al. 2004). Hence, further studies are needed to clarify the background and consequences of CHOP activity in tumors. Taken together, the PERK pathway is an extremely attractive target for cancer therapies. However, despite the fact that the PERK inhibitor (GSK2656157) effectively prevented tumor growth in preclinical models, the toxicity of this molecule against pancreatic β-cells prevented it from reaching the clinic (Atkins et al. 2013; Magnaghi et al. 2013, 2012). This failure of PERK inhibition as a cancer therapeutic was expected since both mice and humans with a genetic loss of PERK suffer from rapid β-cell death (Harding et al. 2001; Zhang et al. 2002). Notably, the integrated stress response inhibitor (ISRIB, in preclinical development), unlike PERK inhibitors, limits the ISR activated by chronic low-level stress, but preserves enough activity to protect against acute stress. In other words, ISRIB rescues translation only if eIF2 phosphorylation is below the threshold and reduces tumor size in transgenic mice and in patient-derived xenografts without provoking type 1 diabetes (Rabouw et al. 2019).

Notably, UPR branches are only partially independent of each other, and therefore the effectiveness of approaches that inhibit the activity of PERK or IRE1 and their off-target effects are difficult to accomplish. For example, prostate cancer cells often have loss of phosphatase and tensin homolog (PTEN) that is accompanied by IRE1-driven MYC hyperactivation (in fatal-metastatic cases) (Sheng et al. 2019). Although such a phenotype would be expected to increase protein synthesis, the overexpression of Myc in a *Pten* knockdown murine model of prostate cancer resulted in a PERK-dependent reduction of translation (Nguyen et al. 2018). It has been also reported that proteasome inhibition is more effective against glioblastoma cells in vitro when accompanied by STK047915, a putative inhibitor of the IRE1–ASK–JNK pathway (Kim et al. 2009). Taken together the complexity of the UPR signaling and cancer specific mutations remain serious obstacles for the development of anticancer therapies.

## The role of IRE1 in cell fate decisions

Ire1 is the only major UPR sensor that is present in budding yeast, plants, and metazoans, and is often referred to as the most conserved UPR branch (Hollien 2013). The original studies identified the UPR function of Ire1 in *Saccharomyces cerevisiae* (Cox et al. 1993; Mori et al. 1993). Ire1 activation allows it to cleave at two specific sites in the mRNA encoding *Hac1* (Mori et al. 1996; Cox and Walter 1996) that removes a regulatory intron from the message to form transcriptionally active Hac1p protein. Hac1p upregulates expression of genes responsible for the secretory pathway as well as BiP (Travers et al. 2000). This pathway is conserved in most eukaryotes and homologous to IRE1-XBP1 branch in mammals (Hollien 2013). Two isoforms of Ire1 have been identified in mammals. Ire1α that we refer to here as IRE1 and is expressed ubiquitously (Tirasophon et al. 1998) and essential for both embryonic development and the UPR (Iwawaki et al. 2009). The second is Ire1β whose expression is limited to intestinal epithelial cells and its deletion sensitizes mice to colitis (Bertolotti et al. 2001). Notably, although both these isoforms can splice *XBP1* mRNA (Calfon et al. 2002), Ire1α is the more efficient, however, Ire1β exerts a stronger RIDD activity (Imagawa et al. 2008).

IRE1 RNase activity is the most prominent activity and has two distinct signaling outcomes, *XBP1* splicing and RIDD induction (Iwakoshi et al. 2003). Furthermore, the cytoprotective effects are mainly related to *XBP1* splicing, whereas apoptosis is RIDD-related (Iwakoshi et al. 2003). Importantly, it has been shown that initially *XBP1* mRNA splicing prevails over the RIDD, but at the time when the maximum levels of XBP1 are reached, RIDD activity increases until apoptosis occurs (Iwakoshi et al. 2003). This suggests that although stress intensity enhances RIDD to favor cell death, this IRE1 activity may have other functions (Iwakoshi et al. 2003). Thus, IRE1 activity with two distinct outcomes indicates IRE1’s important role in determining cell fate during ER stress.

Production of *XBP1s* mRNA is a result of IRE1 catalyzed removal of a 26-nucleotide intron from *XBP1* mRNA and ligation of the remaining fragments by the tRNA ligase RtcB (Yoshida et al. 2001, 2000). The resulting *XBP1s* mRNA, due to the splicing-related frameshift (upon splicing 3’UTR fragment becomes a coding sequence) provides a template for a longer and transcriptionally active XBP1s protein. Thus, the XBP1s protein (~ 48 kDa) has the same N-terminus, but a longer and distinct C-terminus which contains the transactivation domain (Yoshida et al. 2001, 2000). In the absence of IRE1 activity, the unspliced XBP1 protein (~ 29 kDa) is rapidly degraded (Tirosh et al. 2006), while during ER stress, *XBP1* transcription is enhanced by ATF6f (Yoshida et al. 2001, 2000). XBP1s enhances expression of ERAD components (*EDEM1*), chaperones (*HSPA5*, *DNAJB9*, and *DNAJC3*), and vesicle-trafficking components (*SEC23B*) (Bartoszewska et al. 2019, 2017; Gebert et al. 2021; Lee et al. 2003; Misiewicz et al. 2013). The activity of XBP1s cooperates or overlaps with ATF6f, and ATF6f in islet cells has been shown to be necessary to fully activate XBP1s targets. In contrast XBP1s was not required for the activation of ATF6f targets (Sharma et al. 2020). However, genes that contain unfolded protein response elements (UPRE) in their promoter sequences depend solely on XBP1 (Yamamoto et al. 2004). Moreover, XBP1s has been shown to regulate genes involved in the inflammatory responses (Shaffer et al. 2004), as well as genes not related to UPR pathways including adipocyte and myogenic differentiation (*C/EBP* and *MIST1*) in a tissue-dependent manner (He et al. 2010).

In a recent study, we demonstrated that elevating XBP1s expression during ER stress using an inducible cell line correlated with a clear prosurvival effect and reduced PERK-related proapoptotic PUMA protein expression (Gebert et al. 2021). We also identified a novel negative-feedback regulatory loop between XBP1 and IRE1 and showed that XBP1s attenuates *ERN1* expression and thus reduces IRE1 activity, further evidence that XBP1s is crucial for the UPR cell fate decisions (Gebert et al. 2021).

RIDD is IRE1-mediated cleavage of ER-bound RNA (mRNA, miRNA, and rRNA) and was first described in *Drosophila melanogaster* (Hollien and Weissman 2006*),* and later found to be conserved in mammals (Hollien et al. 2009). RIDD was first believed to be a sequence-specific process in which ER-localized mRNAs were cleaved at a consensus motif similar to XBP1 splicing sites (Hollien et al. 2009; Maurel et al. 2014; Tirasophon et al. 2000). The free ends of generated mRNA fragments are then substrates for cellular exoribonucleases that rapidly degrade them (Iqbal et al. 2008). Although a later report questioned the requirement of the splicing motif and suggested RIDD to be a default pathway for ER-localized mRNA (Gaddam et al. 2013). To date, there is no experimental proof that IRE1 is able to cleave at a non-XBP1-like site (Maurel et al. 2014). Nevertheless, several studies have identified RIDD-degraded mRNAs that encode cytosolic or nuclear proteins (including *XBP1*), illustrating that the IRE1 substrates do not necessarily require an ER a signal sequence (Kraut-Cohen and Gerst 2010; Pyhtila et al. 2008; Lerner et al. 2003; Diehn et al. 2000). Importantly, IRE1 has also been shown to degrade several pre-miRNAs (Upton et al. 2012; Gebert et al. 2023) and to facilitate maturation of miRNA precursors in a DICER-independent manner (Avril and Chevet 2020). Because IRE1 is also localized in the inner nuclear envelope (Schroder and Kaufman 2005), these precursors could be processed as they encounter IRE1 while traversing the nuclear pore on their way to the cytoplasm (Upton et al. 2012).

Given that miRNA expression profiles are strongly affected by the ER stress (Gebert et al. 2018; Maurel and Chevet 2013; Bartoszewska et al. 2013), RIDD may indirectly fine tune a variety of UPR outputs at the posttranscriptional level. Mouse miRNAs were shown to be degraded by RIDD and this action was proposed to permit increased expression of caspase-2 (Lerner et al. 2012). Follow-up studies, however, questioned this miRNA-CASP2 mRNA interaction (Sandow et al. 2014). Nevertheless, RIDD-dependent degradation of one of these miRNAs precursors, pre-miR-17, was shown to enhance the expression of the pro-oxidant thioredoxin-interacting protein (*TXNIP*) that led to an inflammatory response-related cell death (Lerner et al. 2012). Although more detailed studies are required to understand miRNA-related consequences of RIDD, this aspect of IRE1 activity seems to accelerate cell death. In support of this, examples of cells under irremediable ER stress indicate that IRE1 becomes hyperactive and besides enhancing RIDD, also serves as a scaffold for the activation of proinflammatory and apoptotic ASK1-JNK and NF-κB pathways (Zeng et al. 2015; Ghosh et al. 2014).

A number of reports have indicated that RIDD activity allows for the degradation of mRNAs encoding growth-promoting proteins and linked to proliferation, and thus lead to cell death (Hetz 2012; Maurel et al. 2014). Since RIDD activity has been also shown crucial for the accelerated cell death of glioblastoma multiform and triple negative breast cancer (Lhomond et al. 2022; Martinez-Turtos et al. 2022), this aspect of IRE1 activity might be considered to be an important candidate for anticancer therapies. However, such a RIDD-oriented approach may be limited by the basal activity of this pathway and potential off-targets effects. In mammals under no stress conditions, RIDD remains active and can serve a cytoprotective role and this is termed basal RIDD (Dejeans et al. 2012; Pluquet et al. 2013; So et al. 2012). Our studies have indicated that while IRE1 RNAse activity supports HIF-1α accumulation in hypoxia exposed human endothelial cells, *XBP1* splicing is absent (Moszynska et al. 2020). Thus, RIDD modulates the adaptive response to hypoxia (Moszynska et al. 2020). RIDD-mediated degradation of transcripts encoding P450 cytochrome variants prevents liver cells from acetaminophen-induced toxicity and modulates proinsulin secretion (Hur et al. 2012; Lipson et al. 2006). Furthermore, IRE1β-related RIDD has been shown to be significantly active in the presence or absence of ER stress. Taking into an account that RIDD activity increases progressively with ER stress intensity/duration, this suggests that UPR is the only mechanism for enhancing RIDD activity (Hollien et al. 2009; Pluquet et al. 2013). Furthermore, the *XBP1* deficient models display enhanced RIDD, suggesting that there is crosstalk mechanism between these two RNase activities (So et al. 2012; Osorio et al. 2014). Taken together, basal RIDD modulates the entry of proteins into the ER in response to the cellular requirements and provides a physiological way of maintaining ER homeostasis (Maurel et al. 2014). Under ER stress, however, basal RIDD is inefficient and therefore IRE1’s XBP1s splicing activity is initiated, whereas RIDD gradually increases. If the stress is too strong or persistent and this fails, RIDD remains hyperactive despite the inactivation of XBP1s signaling and this leads to cell death (Maurel et al. 2014). Taken together, the RIDD activity threshold controls the switch between survival and apoptotic function of this IRE1 activity.

Deregulation of IRE1 signaling, including overactivation of *XBP1* splicing, has been reported as promoting proliferation of several cancer types, including glioblastoma, breast, prostate, and pancreas (Sheng et al. 2019; Chen et al. 2014; Pommier et al. 2018). Notably, this IRE1-related cancer-promoting mechanism is not limited to UPR but also include immunomodulation (Logue et al. 2018; Obacz et al. 2019). In this regard, activation of IRE1 signaling may protect tumors from the immune system by interfering with immune responses (Chen and Cubillos-Ruiz 2021**).** For example, XBP1s has been shown to reduce major histocompatibility complex class I (MHC-I) surface presentation (Almeida et al. 2007) and XBP1s-induced miR-346 has been shown to inhibit MHC-I assembly (Bartoszewski et al. 2011**).** Numerous studies have also connected the altered function of immune cells against cancer cells through XBP1s-mediated expression of the proinflammatory factors (Logue et al. 2018; Obiedat et al. 2019; Chopra et al. 2019; Thevenot et al. 2014; Mohamed et al. 2020; Harnoss et al. 2020; Hurst et al. 2019; Bottcher and Sousa 2018). Breast cancer cells, including triple negative breast cancer, upon pharmacological IRE1 inhibition, display reduced expression of immune modulators such as interleukin 8 (*IL-8*), C-X-C Motif Chemokine Ligand 1 (*CXCL1*), or transforming growth factor-beta 2 (*TGF2*) (Logue et al. 2018). Interestingly, some anticancer drugs like paclitaxel can increase XBP1s levels and the secretion of the above-mentioned cytokines, and lead to restored cancer proliferation following the chemotherapy (Marciniak et al. 2022; Raymundo et al. 2020). This would agree with the report from triple-negative breast cancer mouse xenografts cotreated with both paclitaxel and an IRE1 inhibitor (MKC8866) (Sanches et al. 2014). Although the IRE1 inhibitor was inefficient alone, its combination with paclitaxel delayed the time to tumor regrowth after stopping the treatment (Marciniak et al. 2022; Raymundo et al. 2020). Furthermore, some cetumximab-treated cancer cells that are resistant to immunogenic cell death, display increased *XBP1s* expression, and inhibition of XBP1 splicing restored tumor immunogenicity (Huo et al. 2020; Pozzi et al. 2016).

## Pharmacological targeting of IRE1 in anticancer approaches

Gaining pharmacological control over IRE1 activities has been the focus of several anticancer drug development strategies, and these can be divided into two main approaches: (i) inhibiting IRE1 activity to impair adaptability of tumor cells to challenging tumor microenvironment and (ii) activating or hyperactivating IRE1 to initiate its RIDD that leads to cell death. Furthermore, downstream IRE1 signaling components have also been shown to be of interest to drug discovery programs (Marciniak et al. 2022; Raymundo et al. 2020; Carlesso et al. 2019, 2020; Mahdizadeh et al. 2020, 2021; Doultsinos et al. 2021; Mercado and Hetz 2017; Dufey et al. 2020).

Preventing IRE1 involvement in the cancer UPR with small molecules represents the main course of drug development pipelines. Their goal is to identify compounds targeting either the kinase domain or the RNase domain (Table 1). Since IRE1 phosphorylation is required for the activation of endoribonuclease-based production of proadaptive XBP1s, an ATP-competitive inhibitor such as type II kinase-inhibiting RNase-attenuators (KIRAs) has been shown to be effective in reducing the IRE1 RNase activity (Ghosh et al. 2014; Wang et al. 2012; Papandreou et al. 2011; Morita et al. 2017) (reviewed in Raymundo et al. (2020). Furthermore, other compounds with a different mechanism of action were selected based on the high throughput screening approaches (Doultsinos et al. 2017**).** Many of these molecules are direct inhibitors (including 4μ8C) that specifically target at lysine 907 in RNAse domain and this impairs RNA splicing (Sanches et al. 2014; Sun et al. 2016; Tang et al. 2014; Mimura et al. 2012). Notably one of these compounds, STF-083010, has been shown to block IRE1 endonuclease activity without affecting its kinase activity and it displays selective cytotoxicity towards cancer cells including breast cancer (Papandreou et al. 2011). In contrast to STF-083010, 4μ8C inhibits the IRE1 autophosphorylation by interaction with lysine 599 in the kinase domain (Stewart et al. 2017; Cross et al. 2012). Another class of compounds, salicylaldehydes, was shown to compete against the XBP1 stem-loop RNA substrate (Volkmann et al. 2011). Moreover, although other compounds (including toyocamycin, doxorubicin, quinotrierixin, and trierixin) were reported to inhibit IRE1/XBP1s activity in vitro and in vivo*,* but their mode of action remains unknown (Raymundo et al. 2020).

**Table 1:** Molecules targeting IRE1 (inhibitors and activators)

Compound	Kinase activity	RNase activity	RIDD	Comments	References
Compound 3	Inhibits	Inhibits	?	Type II inhibitor	Carlesso et al. (2018)
				Impairs IRE1 oligomerization	
Compound 6	Inhibits	Inhibits	?	Type II inhibitor	Feldman et al. (2019)
				IRE1α selective	
Compound 15	Inhibits	Inhibits	?	Type II inhibitor	Feldman et al. (2019)
				IRE1β selective	
KIRA6 (compound 3 analogue)	Inhibits	Inhibits	Inhibits	Type II inhibitor	Ghosh et al. (2014); Morita et al. (2017); Mahameed et al. (2019)
				**Preclinical development**	
KIRA7 (compound 3 analogue)	Inhibits	Inhibits	Inhibits	Type II inhibitor	Ferri et al. (2020); Thamsen et al. (2019)
				**Preclinical development**	
KIRA8 (AMG-18)	Inhibits	Inhibits	Inhibits	Type II inhibitor	Ferri et al. (2020); Morita et al. (2017); Morita et al. (2017); Feldman et al. (2016b)
				**Preclinical development**	
AD60	Inhibits	Inhibits	?	Type II inhibitor	Mendez et al. (2015)
				**Preclinical development**	
Compound 31	Inhibits	Inhibits	?	Direct inhibitor	Colombano et al. (2019)
GSK2850163 (GlaxoSmithKline)	Inhibits	Inhibits	?	Type III inhibitor	Concha et al. (2015)
				**Preclinical development**	
STF-083010	No effect	Inhibits	Inhibits	Direct inhibitor	Sun et al. (2016)
HNA	?	Inhibits	?	Direct inhibitor	Sun et al. (2016)
B-I09 (4μ8C analogue)	?	Inhibition of XBP1 splicing	?	Direct inhibitor	Tang et al. (2014)
4μ8C	Inhibits	Inhibits	Inhibits	Direct inhibitor	Cross et al. (2012)
OICR573	No effect	Inhibits	?	Direct inhibitor	Sanches et al. (2014)
OICR464	No effect	Inhibits	?	Direct inhibitor	Sanches et al. (2014)
MKC-3946	No effect on auto-phosphorylation	Inhibits	?	Direct inhibitor	Mimura et al. (2012)
MKC9989	?	Inhibits	?	Direct inhibitor	Sanches et al. (2014)
MKC8866	?	Inhibits	Inhibits	Direct inhibitor	Sanches et al. (2014)
MK018693	?	Inhibits	?	Direct inhibitor	Volkmann et al. (2011)
C-1305	No effect	Inhibits	?	Direct inhibitor	Bartoszewska et al. (2021)
Doxorubicin	?	Inhibits	No effect	Anthracycline antibiotic	Jiang et al. (2016)
3-methoxy-6-bromosalicylaldehyde	?	Inhibits	?	–	Volkmann et al. (2011)
Trierixin	Inhibits	?	?	–	Tashiro et al. (2007)
APY24	Activates	?	?	Type I kinase inhibitor	Mendez et al. (2015)
APY29 (APY24 analogue)	Activates	?	?	Type I kinase inhibitor	Korennykh et al. (2009)
IPA (APY24 analogue)	Activates	?	?	Type I kinase inhibitor	Mendez et al. (2015)
CRUK-3 (originally named compound 3)	Inhibits	Activates	?	Type I kinase inhibitor	Joshi et al. (2015)
G-1749 (KIRA8 analogue)	Activates	?	?	Type I kinase inhibitor	Ferri et al. (2020)
G-9807	Activates	?	?	Type I kinase inhibitor	Ferri et al. (2020)
Sunitinib (Pfizer)	Inhibits	Activates	?	Tyrosine kinase (RTK) inhibitor	Feldman et al. (2016a); Korennykh et al. (2009)
				**In clinical use**	
CXC195	?	Stabilization of IRE1-TRAF2-ASK1 complex	?	Binds IRE1 dimers	Chen et al. (2015)
IXA4	No effect	Activates	?	Requires IRE1 phosphorylation	Grandjean et al. (2020)
				XBP1s specific	
IXA6	No effect	Activates	?	Requires IRE1 phosphorylation	Grandjean et al. (2020)
				XBP1s specific	
Quercetin (flavanol)	Activates	?	?		Wiseman et al. (2010)

Type I kinase inhibitors bind to the active conformation, type II kinase inhibitors bind to the inactive/closed conformation, and type III kinase inhibitors bind next to the ATP site

Some kinase type I inhibitors such as sunitinib have been shown to activate IRE1 RNase activity by promoting oligomerization of this enzyme (Feldman et al. 2016a; Korennykh et al. 2009). Another IRE1 activating compound, CXC195, interacts with cysteine 645 in the kinase domain and leads to increased IRE1 scaffolding activity (Rosen et al. 2019; Chen et al. 2015). Many of the IRE1 targeting compounds were not originally identified as such. For example, sunitinib malate (Pfizer Sutent®) was identified as an oral multi-kinase inhibitor preventing the growth, proliferation, and spread of cancers by targeting vascular endothelial growth factor receptor (VEGFR) and platelet-derived growth factor receptor (PDGFR) (Raymond et al. 2011). In our study, we identified triazoloacridone C-1305, a microtubule stabilizing agent that also has topoisomerase II inhibitory activity, to also be a direct IRE1’s RNase inhibitor (Kroliczewski et al. 2020; Bartoszewska et al. 2021; Switalska et al. 2022). However, the wide range of other activities that many IRE1 targeting compounds have may increase the risk of off-target effects, and thus this may limit their clinical application.

In summary, many attempts have been made to translate IRE1 targeting compounds into anticancer therapies (Table 1). Both treatments with IRE1 inhibitors alone or in combination with other cancer drugs have been shown to be effective against many tumors in both in vitro and in vivo models as well as some in clinical treatments (Table 1) (Logue et al. 2018; Sun et al. 2016; Ri et al. 2012; Jiang et al. 2016; Harnoss et al. 2019). Notably, adjuvant use of MKC8866 was found to be supportive in anti-glioblastoma multiform therapy (Reste et al. 2019).

Furthermore, in recent years numerous natural compounds have been reported to activate UPR-related cell death signaling pathways in different types of cancer cells (reviewed in Limonta et al. 2019). Notably, some of these compounds were reported to affect IRE1 levels and signaling and thus they may provide a starting point for the next generation of IRE1 inhibitors or activators (Table 2). However, further studies to define the pharmacological properties of these compounds as well as molecular mechanisms associated with their impact on IRE1 expression and activity are required before therapeutic approaches can be tested and utilized.

**Table 2:** Natural compounds that affect IRE1 expression or signaling

Compound	Type	Impact on IRE1 activity	IRE1 related mechanism	Comments	References
Curcumin	Polyphenol	?	?	Increased expression of IRE1	Rivera et al. (2017)
Bisdemethoxycurcumin	Polyphenol	?	?	Increased expression of IRE1	Yang et al. (2016)
Demethoxycurcumin	Polyphenol	?	?	Increased expression of IRE1β	Ko et al. (2015)
Resveratrol	Polyphenol	?	?	Increased expression of IRE1	Chow et al. (2014)
(-)-Epigallocatechin-3-gallate	Polyphenol	?	?	Increased levels of XBP1s	Martinotti et al. (2018)
γ-Tocotrienol	Tocotrienols	?	?	Increased levels of XBP1s and DR5	Park et al. (2010); Comitato et al. (2016); Montagnani Marelli et al. (2016)
δ-Tocotrienol	Tocotrienols	?	?	Increased levels of XBP1s and DR5	Park et al. (2010); Comitato et al. (2016); Montagnani Marelli et al. (2016)
Garcinone-E	Xantone	?	?	Increased levels of XBP1s	Xu et al. (2017)
Gambogic acid	Xantone	?	?	Increased levels of *XBP1s* mRNA	Krajarng et al. (2015)
Pristimerin	Terpenoid	?	?	Increased expression of IRE1	Cevatemre et al. (2018)
4-Nerolidylcatechol	Sesquiterpenoid	?	?	Increased expression of IRE1	Alves-Fernandes et al. (2019)
Quercetin	Falvonol	Activates	Activates kinase activity		Wiseman et al. (2010)

Besides IRE1’s potential role in cancer, this enzyme is crucial for human metabolic regulation, and therefore alterations in function can lead to metabolic diseases (Huang et al. 2019) as well as neurological disorders (Marciniak et al. 2022; Vasquez et al. 2022). Indeed, the UPR has been associated with obesity-related metabolic disorders, insulin resistance, and inflammatory responses. IRE1 signaling has also been recognized as crucial for the integration of metabolic stress signals (reviewed in Huang et al. 2019). Thus, pharmacological strategies that aim to restore balance between IRE1s’ XBP1s and RIDD activities are not only limited to anticancer therapies but can also be crucial for effective treatments of metabolic disorders such as diabetes. Nevertheless, gaining specific control over both types of IRE1 activities remains a major challenge for therapeutic strategies. For example, this can be seen in mouse pancreatic islet cells, where both continuous *Xbp1s* overexpression or *Xbp1* knockdown result in impaired insulin secretion, increased RIDD activity, and β cells death (Allagnat et al. 2010; Lee et al. 2011). Basal IRE1 activity is essential for pancreatic islet growth and oxidative stress resistance (Hassler et al. 2015; Tsuchiya et al. 2018; Xu et al. 2014). Taken together, these studies demonstrate that IRE1-related therapeutic approaches that aim to restore β cells homeostasis and increase their ability to produce insulin will require careful modulation of both *XBP1s* splicing and RIDD activity. Furthermore, the importance of UPR is highlighted by the fact that basal IRE1 activity and XBP1s production is required for brain homeostasis, and this suggests a novel therapeutic strategy for aging-related neurodegeration (Krukowski et al. 2020; Cabral-Miranda et al. 2022). However, the complexity of the UPR pathway remains the major challenge of dedicated therapies, limiting the number of dedicated compounds that make it to clinical use (as reviewed in Marciniak et al. 2022). Currently, there is only one marketed IRE1 inhibitor (sunitinib), and the other UPR-related drugs are either specific protein-dedicated pharmacological chaperones (lumacaftor for cystic fibrosis transmembrane conductance regulator (CFTR)) or proteasome inhibitors (like bortezomib, ixazmomib, and carfilzomib) (Marciniak et al. 2022). Hopefully, ongoing clinical trials will expand this list.

## Conclusions

Despite the ongoing research studies on UPR, the complexity of this pathway impedes its straightforward application in anticancer therapies. The molecular crosstalk between UPR branches in both nonmalignant and cancer cells remains understudied and is at this point beyond therapeutic control. The main research barriers include the incredible variability of the different cancers, the complexity of their microenvironments, and how this complexity affects the UPR output signaling.

Even though all the UPR branches provide attractive anticancer therapeutic targets, IRE1 pathways appear to have the most potential given their clear role in cell fate decisions. Since *XBP1* splicing has a prosurvival output, which is often overactive in cancer, it remains a promising target. Targeting RIDD activity, however, with all of its different targets makes it potentially more complex at least at this point. Furthermore, it should not be ignored that the basal RIDD is crucial for maintaining ER homeostasis, and thus further studies of this aspect of IRE1 function are required to minimize the risk of off-target effects. That being said, the identification of compounds that would selectively activate or inhibit a specific aspect of IRE1 RNAse activities is of foremost importance. Finally, although numerous compounds that modulate IRE1 activity are known, their clinical use remains limited (Raymundo et al. 2020), and therefore the search for alternative solutions that will prevent *XBP1* splicing or hyperactivate RIDD are clearly needed.

## Data Availability

Not applicable.
